# Laboratory business models and practices: implications for availability and access to germline genetic testing

**DOI:** 10.1038/s41436-021-01184-z

**Published:** 2021-05-06

**Authors:** Maren T. Scheuner, Michael P. Douglas, Paloma Sales, Sara L. Ackerman, Kathryn A. Phillips

**Affiliations:** 1 Center for Translational and Policy Research on Personalized Medicine (TRANSPERS), Department of Clinical Pharmacy, University of California–San Francisco, San Francisco, CA, USA.; 2 Department of Medicine, Division of Hematology-Oncology and Department of Pediatrics, Division of Medical Genetics, University of California–San Francisco, School of Medicine, San Francisco, CA, USA.; 3 San Francisco VA Health Care System, San Francisco, CA, USA.; 4 UCSF Helen Diller Family Comprehensive Cancer Center, University of California–San Francisco, San Francisco, CA, USA.; 5 Northern California Institute for Research and Education, San Francisco, CA, USA.; 6 Department of Social and Behavioral Sciences, University of California–San Francisco, San Francisco, CA, USA.; 7 Philip R. Lee Institute for Health Policy Studies, University of California–San Francisco, San Francisco, CA, USA.

## Abstract

**PURPOSE:**

Germline testing laboratories have evolved over several decades. We describe laboratory business models and practices and explore their implications on germline testing availability and access.

**METHODS:**

We conducted semistructured interviews with key informants using purposive sampling. We interviewed 13 key informants representing 14 laboratories. We used triangulation and iterative data analysis to identify topics concerning laboratory business models and practices.

**RESULTS:**

We characterized laboratories as full-service (FSL), for-profit germline (PGL), and not-for-profit germline (NGL). Relying on existing payer contracts is a key characteristic of the FSL business models. FSLs focus on high-volume germline tests with evidence of clinical utility that have reimbursable codes. In comparison, a key business model characteristic of PGLs is direct patient billing facilitated by commodity-based pricing made possible by investors and industry partnerships. Client billing is a key business model characteristic of NGLs. Because many NGLs exist within academic settings, they are challenged by their inability to optimize laboratory processes and billing practices.

**CONCLUSION:**

Continued availability of, and access to germline testing will depend on the financial success of laboratories; organizational characteristics of laboratories and payers; cultural factors, particularly consumer interest and trust; and societal factors, such as regulation and laws surrounding pricing and reimbursement.

## INTRODUCTION

Molecular genetic testing for heritable genetic variants (i.e., germline testing) is important to decision-making across all medical specialties, and increasingly individuals are using this genetic information to inform their health, reproductive, and life planning decisions.^1–4^ Germline testing and the laboratories that perform this testing have undergone considerable change in the past several decades due in large part to technological innovations, regulatory requirements, and the 2013 United States Supreme Court ruling invalidating gene patents.^5–11^

Laboratories that offer germline testing are a heterogeneous group.^12,13^ Molecular germline testing first became available in the 1970s, and in the United States, it was performed initially in academic medical centers in clinical or grant-supported research laboratories.^12^ Some laboratory experts at academic institutions created spinoff, for-profit testing laboratories that were quickly acquired by centralized full-service commercial laboratories.^12^ In 1980, a third option emerged: standalone, national commercial laboratories with a focus on germline testing services.^12^

Demand for germline genetic testing has increased exponentially over the decades and is expected to continue to increase. The number of laboratories that perform germline testing has grown as well.^9^ The objective of this work is to describe laboratory business models (the operational plan to market germline tests) and practices (the strategies to support the business plan) and explore the implications of laboratory business models and practices on germline testing availability and access. We defined availability as the supply of germline tests (i.e., performed by a laboratory and included on the test menu) and access as the opportunity to obtain germline tests.^14^

**Fig. 1:**
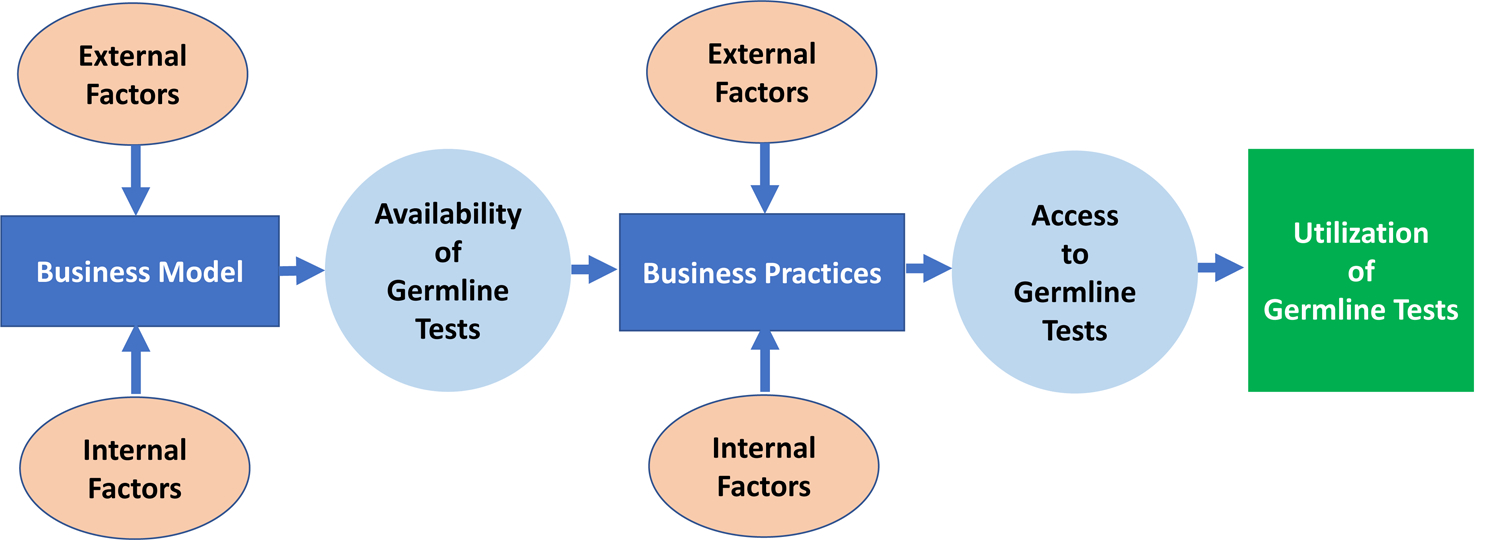
Conceptual model representing key concepts linking laboratory business models, business practices, and availability of and access to germline testing. As depicted, the conceptual model shows the laboratory business model, defined as the operational plan to market germline tests, influences the availability of germline genetic testing. We defined availability as the supply of germline tests (i.e., performed by a laboratory and included on the test menu). Factors external to the laboratory that influence laboratory business models include payer coverage and reimbursement policies; reimbursed Common Procedural Technology codes; testing volume; competition; complexity of the health insurance industry; consumer interest; evidence of clinical utility of germline testing; regulation; labor unions; and shareholders. Factors internal to the laboratory that influence the business model include laboratory structure (e.g., free standing vs. integrated within an organization); ability to automate and optimize processes; efficiencies of scale; culture/philosophy to generate the evidence showing clinical utility of germline testing; and philosophy regarding data sharing. The laboratory business practices, defined as the strategies to support the business plan, influence access to germline testing. We defined access as the opportunity to obtain germline tests. Factors external to the laboratory that influence business practices include germline testing volume; prior authorization requirements; payer requirements for reporting germline results; availability of the genetics workforce; and labor unions. Factors internal to the laboratory that influence business practices were related to the laboratory setting, e.g., the academic setting constrained pricing and hiring of staff. Utilization of germline tests occurs when there is uptake of germline testing by individuals.

This work was informed by a conceptual model representing key concepts linking laboratory business models, business practices, and availability of, and access to germline testing (Fig. 1). As depicted, a laboratory’s business model informs the germline genetic test menu offered by the laboratory, and thus, the availability or supply of such testing in the marketplace. The business practices of laboratories impact opportunities to access these tests. Utilization occurs when there is uptake of germline testing by individuals.

## MATERIALS AND METHODS

### Sample

We conducted semistructured interviews with individuals who were knowledgeable and able to generalize about the laboratories where they have worked (i.e., key informants).^15^ We interviewed 13 key informants representing 14 laboratories. We identified key informants using purposive sampling. We contacted current or former leaders at laboratories that perform germline testing with a goal to have roughly equal numbers from academic and commercial laboratories. Additionally, using a snowball sampling approach, key informants identified additional individuals potentially knowledgeable about the subject. We assured participants of anonymity with the hope this would promote openness in their responses. The institutional review board at the University of California–San Francisco approved all study procedures.

### Data collection

The conceptual model (Fig. 1) helped inform data collection and analysis. All interviews were conducted by the lead author (M.T.S.) and one or two secondary interviewers (M.D., K.P.) using a semistructured guide (see Supplemental Materials). Interview domains included laboratory business models and their evolution, laboratory business practices, factors influencing laboratory business models and practices, and research needed to address germline testing availability and access. We emailed key informants a study information sheet that included items to be discussed and definition of terms. During the interview, we restated definitions. We asked key informants about the germline testing laboratories they have been affiliated with and their role, and their current positions if no longer affiliated. Interviews were conducted between January and July 2020. Interviews were recorded and professionally transcribed. Additionally, comprehensive notes were prepared by the interviewers.

### Data analysis

The interview transcripts were reviewed and edited for accuracy. The transcripts and field notes served as the source of data for a rapid assessment process characterized as an intensive, team-based qualitative inquiry using triangulation and iterative data analysis to develop an understanding of the research findings.^16^ A summary template for the interviews was created with each interview question listed. One team member (M.T.S.) mapped key concepts and representative quotes found in each interview to the summary template. A second researcher (M.D.) reviewed the summaries for accuracy and completeness. Discrepancies in mapping of concepts in the summaries were discussed among the team until consensus was reached. One researcher (M.T.S.) then transferred the data from the summaries into multiple matrices with columns representing interview domains and rows representing responses for each key informant. The matrices helped to streamline the process of noting simultaneously and systematically the similarities, differences, and trends in responses across key informants. Memos were then prepared to describe the key themes identified in analyzing the matrices.

## RESULTS

We grouped the 14 laboratories according to the breadth of services offered, i.e., full-service laboratories versus laboratories that focus mainly on germline testing. Among the germline testing laboratories, we also considered their status as for-profit versus not-for-profit, as this characteristic appeared important in distinguishing these laboratories. Thus, we characterized the laboratories into three types:

- Full-service reference laboratories (FSLs) that include germline testing within their test menus and are for-profit or not-for-profit (*n* = 3).
- For-profit germline testing laboratories (PGLs) (*n* = 5).
- Not-for-profit germline testing laboratories (NGLs) based at academic institutions or biotechnology companies (*n* = 6).

We spoke with chief medical officers, chief operating officers, laboratory directors, medical directors, and administrative directors. Nine had genetics training, including three clinical geneticists, four clinical molecular geneticists, and two molecular genetic pathologists. Two had experience working in the biotechnology/life sciences industry, two had experience working in the health insurance industry, and two had experience with the health technology assessment industry. None worked for a direct-to-consumer (DTC) laboratory. Three key informants worked for multiple germline testing laboratories represented in our sample, either concurrently or formerly. Two worked at the same laboratory.

### Topics

**Fig. 2:**
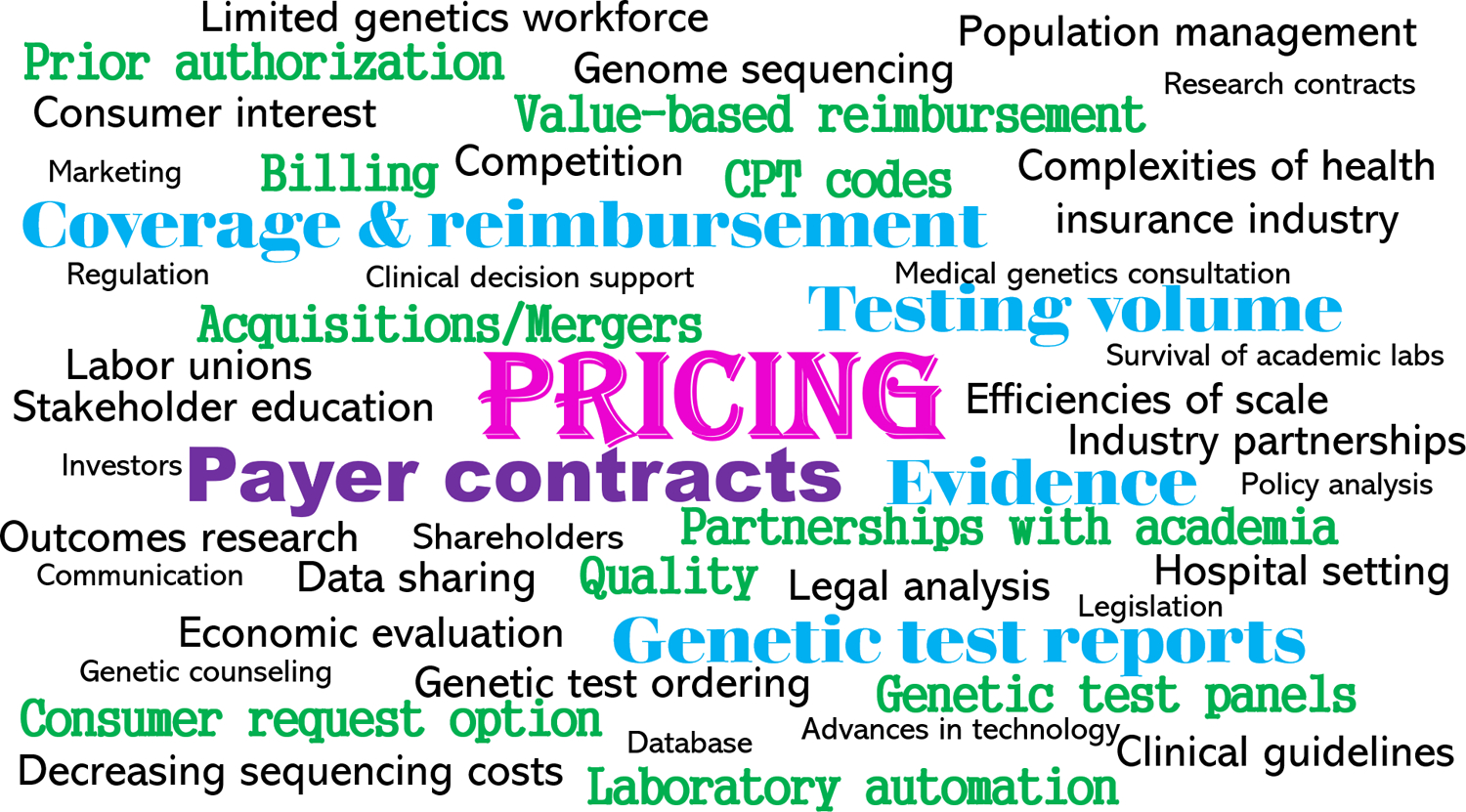
Word cloud illustrating the 48 unique topics identified. The size of font is directly proportional to the number of times a topic was mentioned across the six domains assessed. The domains included (1) laboratory business models, (2) factors influencing laboratory business models, (3) laboratory business practices, (4) factors influencing laboratory business practices, (5) the evolution of business models, and (6) research ideas to improve availability of and access to germline testing. Pricing was most common topic, followed by payer contracts, coverage and reimbursement, testing volume, evidence (of clinical validity, clinical utility, personal utility, and economic value), and genetic test reports.

Figure 2 depicts a word cloud illustrating the 48 unique topics identified. We mapped the topics to the six interview domains. For each topic and corresponding domain, we noted whether one or more key informants from each laboratory type made comments tied to the topic. The most common topic was *pricing* mapping to the four domains of business model characteristics, business practices, factors influencing business practices, and evolution of business models. This was followed by topics of: payer contracts (mapping to business model characteristics, business practices, evolution of business models); payer coverage and reimbursement (mapping to factors influencing business models, evolution of business models); testing volume (mapping to factors influencing business models and practices); evidence of clinical validity and utility (mapping to factors influencing business models, research needed); and genetic test reporting (mapping to business practices, research needed).

### Germline genetic test menus

Generally, FSLs offer germline testing for common, prevalent conditions, such as genotyping of common variants (e.g., factor V Leiden, *HFE* C282Y and H63D) and multigene panels for hereditary disorders that are limited to certain common diseases (e.g., breast cancer). A FSL key informant described decisions about making germline testing available, “Because we are a large reference lab and want to have broad services, leadership may make a decision to be a full-service facility…even if it may not be profitable.” PGLs have test menus that include both common and rare diseases with many multigene panels of varying sizes (often customizable), and clinical exomes. Key informants from NGLs often described filling gaps in germline testing not available at other laboratories. While NGL test menus often include genotyping for common variants, they tended to have more in common with the PGLs. NGLs offer testing for rare disorders with multigene panels, clinical exome, and clinical and research genome sequencing. They also use techniques and methods not readily available elsewhere (e.g., RNA isolation and sequencing, single cell sequencing, epigenetic assays). Other similarities between the PGLs and NGLs include having leadership with genetics training and describing their laboratories as having state-of-the-art expertise in molecular and clinical interpretation. Additionally, pharmacogenetic testing and tumor testing were offerings available at some PGLs and NGLs.

### Laboratory business models

Table 1 shows the characteristics of laboratory business models described by the key informants. Characteristics of all three laboratory types include third-party billing, contracting with health-care organizations/hospitals, and partnerships with academia. FSLs reported gaining expertise through academic partnerships (e.g., develop esoteric tests, interpretation of genetic test results). For example, one FSL key informant stated, “That is the challenge. Who will develop the tests? Maybe academic centers? [We] want to partner with those Centers of Excellence. Partnering with [us] provides huge access to the testing market.” In comparison, PGLs and NGLs more often pursue academic partnerships to foster research endeavors.

Typical business model characteristics of PGLs included business agreements with investors, partnerships with the pharmaceutical and biotechnology industries to develop and market companion diagnostics and pursue gene discovery, commodity-based pricing, third-party and direct patient billing, and consumer-facing services (i.e., germline test orders by laboratory-contracted physicians).

Business model characteristics for NGLs generally included client billing (i.e., contracts to perform testing for other laboratories or health-care organizations that have third-party or direct patient billing arrangements) and contracts to conduct research. Direct patient billing was generally not available for NGLs. Similarly, consumer-facing services were generally not available for NGLs, with some key informants opposed to this. One stated, “I have many, many problems with it. The first one is ethical, that only wealthy people have access to it…. Second, there are so many variants I don’t know how to interpret without a phenotype or at least a family history.”

#### Business model external influencing factors.

Payer coverage and reimbursement policies, availability of Common Procedural Technology (CPT) codes that are reimbursed by payers, and testing volume are factors influencing the business models of all three laboratory types. Regarding reimbursable codes, a FSL key informant stated, “We do an analysis of the current reimbursement landscape. Anything new won’t have a code or reimbursement rate…. [F]or those with payment value, we look at coverage policies and reimbursement from payers.” FSLs and PGLs concentrate on high-volume tests, whereas, NGLs have leveraged the unfilled space of low-volume testing for rare diseases. One NGL key informant stated, “The only way to survive is to do esoteric testing…there are [laboratory] people that have broad knowledge but not that deep esoteric knowledge, and vice versa. Sometimes you need a mix. It is harder in large laboratories. It is hard to know everything.” Competition and the complexity of keeping informed about individual policies of health insurers are factors influencing business models for both PGLs and NGLs. Consumer interest in germline testing was mentioned as another factor influencing the business model of these laboratories. Both FSL and NGL key informants referred to evidence of clinical utility for germline testing as a factor influencing their business models and both rely on payers that value and reimburse genetic tests with robust evidence of clinical utility. Regulation and labor unions were mentioned as challenges influencing the business models of NGLs that are typically located within academic institutions. Shareholders were mentioned as influencing the business models of the FSLs and PGLs.

#### Business model internal influencing factors.

The ability to automate and optimize laboratory processes influence the business models of FSLs and PGLs. These factors are related to the business model characteristic of pursuing high-volume tests. FSLs capitalize on the structure of their overall laboratory business to bring on germline testing, whereas PGLs develop structures and processes specific to germline testing. Conversely, NGLs located within academic medical centers find the structure of this setting restrictive to billing and contracting options. For example, one NGL key informant said, “Because our Tax ID is the same as the hospital, billing has to happen through the contracted rates with the hospital.” Creation of evidence demonstrating the clinical utility of germline testing and data sharing were described as factors influencing (or driving) the business models of PGLs and NGLs. Creating databases to inform interpretation and to support the potential to monetize the genetic data collected was described as another influencing factor for the business model of PGLs. On this topic, one PGL key informant stated, “They want to get you engaged, get your DNA, and then keep you engaged, and get as much information about you as they can so that they can add you to the big genotype–phenotype database and use that for faster, better, cheaper drug discovery and development. So that’s the behind the scenes business model.”

### Business practices

Topics identified regarding laboratory business practices are shown in Table 2. Providing high quality services was a theme for all three types of laboratories. The provider-focused practice of offering assistance with the prior authorization process was mentioned by all laboratory types but to varying degrees. PGLs typically take on the entire prior authorization process. Low- and no-cost pricing, consumer/patient-focused strategies (e.g., free laboratory-based genetic counseling), marketing and communication characterize the business practices of PGLs. One PGL key informant commented about the websites of PGLs, “Because they’re driven by the need for engagement, and to delight the consumer, their interface is very playful.”

#### Business practices external influencing factors.

Most factors influencing laboratory business practices are external to the laboratory. Testing volume was mentioned by key informants from PGLs, which in general have business practices that aim to reduce barriers to testing and understanding results (e.g., low- or no-cost pricing, no-cost genetic counseling). FSLs and PGLs have been influenced by payer requirements including prior authorization and reporting on results for only certain genes with FSLs typically limiting test offerings according to payer requirements and PGLs offering those tests and more, including genes with preliminary evidence of associations with disease phenotypes. One PGL key informant summed this up by stating, “Payer coverage has not been a major factor. We focus on what do the providers want, and where is there a need and volume.” Pricing by NGLs was influenced by labor unions for medical technologists that increase the cost of testing. A NGL key stakeholder described this as follows, “Unfortunately, my cost basis is quite high, typically 60–70% of the costs are labor. Labor is represented by unions, and therefore, extremely expensive.” The limited genetics workforce was cited by a NGL key informant as a challenge to implementing laboratory business practices.

#### Business practices internal influencing factors.

Constraints of the academic setting are factors for NGLs, in particular financial barriers to hiring desired staff (e.g., a genetic counselor for the laboratory) and the limited ability to negotiate on price or to have lower, self-pay pricing.

### Evolution of laboratory business models: the past

The key informants from both PGLs and NGLs provided the most insight regarding how their business models evolved over time and where they envision their business models going in the future. One NGL laboratory director summed things up by stating, “The [germline testing] marketplace has expanded so much. It used to be that all of us, as clinical lab directors, had pretty much the same experience, that’s not how it is anymore.” Important milestones mentioned by several key informants include the 2013 United States Supreme Court decision to invalidate gene patents that helped to “democratize access to genetic testing”; advances in testing technology with subsequent decreasing costs of testing; advances in automation in the laboratory with process optimization; and PGLs entering the market with subsequent price erosion. The NGL key informants had concerns that the new specific CPT codes for genetic tests, rather than stacking of CPT codes, will lead to reductions in reimbursement. NGL key informants also described the business model and practices of PGLs as not sustainable given the reliance on external support from investors or partnerships that expect a return on their investments.

### Evolution of laboratory business models: the future

Key informants from both PGLs and NGLs believe genome sequencing will supersede many genetic tests (e.g., multigene panels, exomes), and through partnerships with health systems and payers, genome sequencing will support genome-based population health management and research. One NGL key informant stated, “I hope long term we move to examine probably genome sequencing for everything and everyone. We need to think about the engagement with a genome over a person’s lifetime that includes both use of that genome for clinical care and for research.” A PGL key informant had similar ideas, “Where we would like to get to, is expand the amount of information we can generate at the outset. We can sequence an individual’s genome and generate reports, as needed, and as they become relevant. Less of a transaction by transaction and more utility oriented…. Kind of a genome management idea.”

PGL key informants see continued growth in testing volume underlying and driving their future business models and practices. Many mentioned consolidation and acquisition of laboratories “to increase the volume needed to survive” and to diversify product offerings in the genetic testing marketplace. Many mentioned partnering with pharmaceutical companies to conduct research in rare disease therapeutics and develop companion diagnostics. These laboratories also see a future more reliant on payers. One PGL key informant said, “What we would hope to see over time are models where, for example, we would move from fee-for-service per test to either value-based care models or [per member per month] with insurance companies.” Some mentioned a move toward a DTC model.

NGL key informants had a very different view of their future business models. Generally, they had concerns about their ability to survive, and will only be able to do so if they can offer “something esoteric” that other types of laboratories cannot do. Rather than client billing, they see switching to payer contracts to control costs and out-of-pocket expenses for patients. One NGL key informant mentioned “the targeted market for sustainability is the payer.” To reduce overhead costs, some mentioned developing vendor contracts for exome/genome sequencing and providing the interpretation only. Some have concerns regarding the sustainability of the genetics workforce, which relies heavily on academic laboratories to train laboratory and clinical geneticists. One NGL key informant stated, “because most everything is going to come out of a reference laboratory, and they won’t do the training.”

### Research agenda to promote germline testing availability and access

Table 3 describes research ideas from the perspectives of the key informants. Most often mentioned was the need for outcomes research to inform the clinical validity, clinical utility, and personal utility of germline testing (particularly exome sequencing, genome sequencing, and polygenic risk scores) for patients and their family members. Demonstrating the value of germline genetic testing and economic evaluations to study the costs and cost-effectiveness of germline testing, particularly genome sequencing compared to other testing, was also frequently mentioned.

## DISCUSSION

We defined three types of germline testing laboratories according to their business models and practices. We found the breadth of germline testing available from laboratories is highly dependent upon their business model, that business practices can promote or hinder access to germline testing, and factors internal and external to the laboratory can influence the business models and practices in similar or different ways depending on the laboratory type.

The laboratory types we defined closely resemble the types in a review of the history of germline testing laboratories, which described commercial national laboratories (FSLs), academic laboratories (NGLs), “hybrid” academic for-profit spinoffs (PGLs), and standalone genetic testing laboratories with national clientele (PGLs).^12^ Phillips et al. defined laboratory types given the emergence of DTC laboratories, comparing them with “traditional” laboratories requiring orders placed by a patient’s provider (FSLs and NGLs) and “hybrid” laboratories offering consumer-facing services (a characteristic of PGLs) that facilitate patient test requests by laboratory-contracted physicians.^17^ However, these prior works did not assess germline test availability and access according to laboratory type.

Payer contracts and billing are business model characteristics important to the availability of germline testing for all laboratory types, but in varying ways. Existing payer contracts are the mainstay for supporting germline testing at FSLs. Consequently, their germline test menus focus on high-volume tests with evidence of clinical utility and reimbursable CPT codes. FSLs rely on their clinical laboratory structures and processes and acquire the necessary expertise to offer germline testing. The PGL business model is less reliant on payers. Investors and industry partnerships permit commodity-based pricing that facilitates direct patient billing with tiered pricing that helps them achieve desired testing volume. Through their industry partnerships, PGLs plan to grow the evidence base for germline tests. As a result, PGLs tend to offer a breadth of germline tests, including tests with preliminary evidence of clinical validity and limited utility. NGLs rely on client billing arrangements, and like the experience of FSLs, their clients value genetic tests with evidence of clinical utility and reimbursable CPT codes. NGLs are important sources of germline testing expertise (test development and interpretation) and research opportunities. They tend to offer esoteric testing for rare disorders using novel techniques. Because many exist within an academic hospital setting, they are challenged by their inability to automate and optimize laboratory processes compared to FSLs and PGLs.

All three laboratory types have business practices promoting quality services. The PGLs have been most adept at creating and responding to market demand for germline testing. However, some key informants raised concerns about ethical and legal issues pertaining to the business practices of PGLs, such as consumer-facing services facilitating test orders through laboratory-contracted physicians and creating databases for the purpose of monetizing genetic information. Others called for standards and transparency in pricing. PGL business practices primarily aim to reduce barriers to accessing germline testing. As a result, the PGLs have introduced considerable competition in the germline testing market, especially for the NGLs. NGL key informants had concerns about continued price erosion introduced by the PGLs and their ability to survive. The loss of NGLs could stifle the training opportunities essential to grow the genetics workforce (already too small to meet the demand)^18,19^ leading to a loss of expertise for test development, research, and clinical innovation. Multiple NGL and FSL key informants pondered the long-term sustainability of the PGL business practices of low- and no-cost pricing. This is also a concern of genetics professionals providing cancer genetic services in California and North Carolina, whose uninsured and underinsured patients rely on their reduced pricing to gain access to germline tests.^20^

The key informants described genome sequencing as the future for germline testing, which has the potential to supplant most existing germline tests. We heard this technology could promote a new business model of genome-based population health management. This would coincide with greater reliance on payers through value-based reimbursement or per-member-per-month contracts. However, evidence demonstrating the clinical utility and economic value of germline testing will be needed for these payer arrangements. Continued partnerships with industry and academia can support the research necessary to generate this evidence. However, partnerships with payers and health-care systems will be important to obtaining the outcomes needed for demonstrating the clinical utility of germline testing. If value-based payer arrangements dominate the future business model of PGLs, it is likely opportunities for no- and low-cost pricing will decrease resulting in reduced access for uninsured and underinsured patients.^21,22^

Our study has limitations that deserve mention. We were not able to ascertain the impact of business models and practices on actual test availability or access from the perspectives of those seeking testing, including providers and patients. Additionally, we could not speak with key informants for all laboratories of the FSL, PGL, or NGL types; thus, our findings are not comprehensive and are limited to the experience in the United States. Lastly, we did not speak with key informants from DTC laboratories, and according to our scheme, we would have included them as a subset of the PGLs.

In summary, our findings suggest we are on the threshold of a disruption in the germline testing marketplace. Continued availability and access to germline testing will depend on the financial success of laboratories, organizational factors of laboratories and payers, cultural factors, particularly consumer interest and trust, and societal factors, such as regulation and laws surrounding pricing and reimbursement. The conceptual model we created can serve as the basis for a framework to inform stakeholder discussions, future research agendas, and policy decisions relating to germline testing availability and access.

## Supplementary Material

Supplemental Materials - Interview Guide

## Figures and Tables

**Table 1.** Laboratory business model characteristics

Emergent topics & sub-topics	For-profit and not-for-profit full-service laboratories (FSL)	For-profit germline laboratories (PGL)	Not-for-profit germline laboratories (NGL)
**Billing**			
Third-party billing^a^	**√**	**√**	**√**
Client billing^b^			**√**
Direct patient billing		**√**	

**Contracting**			
Payers^c^	**√**	**√**	
Healthcare organizations/hospitals	**√**	**√**	**√**
Value-based reimbursement^d^		**√**	
Research^e^			**√**

**Business arrangements**			
Acquisitions/mergers	**√**	**√**	
Investors		**√**	

**Partnerships**			
Industry^f^		**√**	
Academia^g^	**√**	**√**	**√**

**Pricing**			
Diagnostic service	**√**	**√**	**√**
Commodity-based^h^		**√**	

**Consumer facing service** ^i^			
Available		**√**	
Not available	**√**		**√**

a The payer, an insurance company or health agency uninvolved in the direct care of the patient that pays for the care or services rendered to the patient (i.e., the first party).

b Described as ‘account’ or ‘pass-through’ billing, where medical providers compensate a laboratory for testing services and submit claims to payers for those services.

c Payers as described by key informants: health insurance companies, employer groups.

d Reimbursed based on cost-effective, improved quality of care rather than volume.

e Contracts to perform germline testing, most often exome or genome sequencing, to support research performed by other organizations (e.g., biotechnology companies, pharmaceutical companies, or academic institutions).

f Partnerships with pharmaceutical and biotechnology companies for companion diagnostics and gene discovery.

g FSLs tend to gain technological expertise (test development, results interpretation) through academic partnerships, whereas PGLs and NGLs typically have research partnerships with academia.

h Discounted pricing with the expectation of future payoffs.

i Consumers request genetic testing using a laboratory contracted physician or medical corporation.

**Table 2.** Laboratory business practice characteristics

Emergent topics & sub-topics	For-profit and not-for-profit full- service laboratories (FSL)	For-profit germline laboratories (PGL)	Not-for-profit germline laboratories (NGL)
**Quality services**			
Preferred provider status^a^	√	√	
State-of-the-art, first-in-class services		√	√

**Pricing**			
Premium pricing	√	**√** ^b^	√
Low- and no-cost pricing		√^c^	

**Provider-Focused Strategies**			
Assist with prior authorization	**√**	**√**	
Easy-order web-based portals		√	
Tailored genetic test reports		√	
Provide medical genetics expertise		√	√

**Consumer/Patient-Focused Strategies**			
Website engagement with ongoing feedback		√	
No-cost post-test genetic counseling		**√**	
Offer medical genetics consultation		√	

**Marketing Strategies** ^d^		**√**	

**Communication Strategies** ^e^		**√**	

a Meets payers’ criteria for genetic testing enabling the laboratory to avoid prior authorization requirements with possible auditing by the payer periodically

b Only one PGL key informant mentioned premium pricing

c Low-cost with cash/self-pay, no-cost options for research testing or hardship cases, or no cost for cascade testing in family members.

d Examples: Sales team differentiates products from competitors; marketing to non-genetics providers; payers market division for contracting

e Examples: Media/news releases; communicating with technology assessment groups; communicating with payers about their coverage policies

**Table 3.** Key informant suggestions for research needed to improve availability of and access to germline testing

Research suggestions	Example suggestions	For profit and not-for-profit full-service laboratories (FSL)	For-profit germline laboratories (PGL)	Not-for-profit germline laboratories (NGL)
Building genetic test panels	Genes to be reported on a panel; building appropriate test panels	**√**	**√**	**√**
Reporting genetic test results	Need structured genetic health reports in electronic health records so genetic information can be consumed	**√**		**√**
Demonstrate clinical validity and utility	Demonstrate value of germline testing; demonstrate clinical utility; how to aggregate data to help inform clinical utility; utility of genetic testing for different clinical scenarios and conditions; compare diagnostic yield of genetic testing vs. other diagnostics; polygenic risk scores clinical validity and utility		**√**	**√**
Demonstrate personal utility	Show patient and family perceived utility			**√**
Outcomes research	Partnerships to achieve long-term outcomes; outcomes research for exome		**√**	**√**
Develop study designs	Convene stakeholders to identify evidence gaps and create study designs to fill evidence gaps; need study designs for rare diseases		**√**	**√**
Economic evaluation	Cost analyses/cost-effectiveness of genetic testing; cost-effectiveness of genome compared to other testing; clinical utility and cost savings (longer term studies), show economic benefit from testing; examine how demand for economic utility benefits/harms patients		**√**	**√**
Develop clinical guidelines	Harms/benefits of strict guidelines for genetic testing, for number of genes on panel; focus on guideline development for categories of genetic disorders to inform care pathways instead of rare individual conditions; make sure guideline committees make clear recommendations, don’t use works like “suggest” or “consider” but “recommend”		**√**	**√**
Legal analysis of genetic test pricing	Standardization and transparency in pricing; legal analysis of two-tier pricing		**√**	**√**
Policy analysis of payer coverage	Payer medical policy development and analysis; how to align Medicare and Medicaid; how to influence payer coverage decisions		**√**	
Policy analysis of genetic test regulation	Impact of legislation and FDA regulation on lab developed tests; transparency in molecular test coding		**√**	
Educate stakeholders	Educate non-genetics providers; educate Centers for Medicare and Medicaid policymakers and commercial payers		**√**	

## Data Availability

Materials and data from this work will be supplied upon request.
