# Supplementary material for: Laboratory business models and practices: implications for availability and access to germline genetic testing
Source: Genet Med. Author manuscript; Available in PMC 2022 Mar 1. (PMC8460432; doi:10.1038/s41436-021-01184-z)
Supplement: Supplemental Materials - Interview Guide [file NIHMS1708811-supplement-Supplemental_Materials_-_Interview_Guide.docx]

# Supplemental Materials – Interview Guide

**Introduction:** We are speaking with key informants from molecular genetic laboratories to understand how coverage policies and reimbursement practices of health insurers have influenced laboratory business models and practices, and whether certain laboratory models and practices influence genetic test access and utilization.

**Background**: I would like to begin by getting some background information from you. In researching your laboratory, we found that it performs germline genetic testing for [insert conditions] using [insert testing methods here].

1. Is this an accurate description? Is there anything else you would like to add?

*[When we cannot find information]: What kinds of germline genetic tests do you offer (e.g., single gene, gene panels, exomes, genomes) and for what types of conditions?*

1. I understand you are the [insert text describing position] at [inset name of laboratory]. Is that correct? How long have you worked at [insert name of laboratory]. [*When position not known] What is your current position at the laboratory and how long have you worked there?*

**Business model**: The next set of questions pertain to your laboratory’s business model, that is, the plan in place to support your laboratory operations for germline genetic testing.

1. Can you describe your laboratory’s business model? For example:
   1. Is your laboratory best described as for-profit or not-for-profit?
   2. Have you formed any partnerships with other entities, such as healthcare organizations, health informatics organizations, or research organizations?
   3. Are you supported by philanthropy?
   4. Are you supported by licensing agreements?
2. What factors have influenced the development of your current business model?
3. How has your business model evolved in the past five years?
4. How does your business model influence the types of genetic tests offered by your laboratory? For example, the type of tests performed or the conditions tested?

**Business practices**: Next, I would like to ask about your laboratory’s business practices or market share strategies that your laboratory uses to increase your market share and improve access to genetic testing. I noticed on your website that you offer [insert items, such as prior authorization verification, cash payment options, hardship payment options, and no- or low-cost cascade testing for family members].

1. Is this list accurate? Does your laboratory have any other business practices that come to mind? *OR, if not listed on website, Can you tell me about the business practices your laboratory uses to improve access to genetic testing?*
2. How have coverage policies and reimbursement practices of health insurers influenced your current business practices?
3. What other factors have influenced the development of your current business practices?
4. How have your business practices evolved in the past five years?
5. Which business practices have been most effective in reducing barriers to accessing genetic tests by your customer base?
6. What else could your laboratory do to make genetic testing more accessible?
7. Do you know if your business practices result in more appropriate or guideline-concordant genetic testing?
8. What could be done by others, such as healthcare organizations, professional societies or governmental agencies to improve access to appropriate genetic testing?
9. How does your laboratory assess the effectiveness of your business practices?
   1. What kinds of analyses are performed?
   2. Are these analyses published or publicly available?
   3. Can you compare your results to other laboratories?

**Research interests**: As mentioned previously, we are interested in studying factors influencing access to and utilization of germline genetic testing. We want to understand how coverage policies and reimbursement practices of health insurers impact laboratory business practices, and how those business practices influence genetic testing access and utilization.

1. Has your laboratory assessed the influence of payment policies on genetic test utilization or uptake by patients you serve?
   1. For example, does your laboratory monitor how often the prior authorization process results in denial? The reason for those denials? What actions can successfully reverse those denials and how often does this occur? And how often do patients pay out-of-pocket after a denial?
   2. Does your laboratory assess what patient, ordering provider and insurance characteristics are associated with denial of germline genetic testing?
2. What other factors have influenced genetic test utilization by your customer base?
3. In considering our research interests, what research questions do you think are important to address?
